# Mortality patterns over a 10-year period in Kibera, an urban informal settlement in Nairobi, Kenya, 2009–2018

**DOI:** 10.1080/16549716.2023.2238428

**Published:** 2023-07-25

**Authors:** Clifford Oduor, Irene Omwenga, Alice Ouma, Robert Mutinda, Samwel Kiplangat, Ondari D. Mogeni, Leonard Cosmas, Allan Audi, George S. Odongo, David Obor, Robert Breiman, Joel Montgomery, George Agogo, Patrick Munywoki, Godfrey Bigogo, Jennifer R. Verani

**Affiliations:** a Centre for Global Health Research, Kenya Medical Research Institute (KEMRI), Nairobi, Kenya; b Epidemiology, Public Health, Implementation & Clinical Development Unit, International Vaccine Institute (IVI), Seoul, South Korea; c Centre for Global Health Research, Kenya Medical Research Institute (KEMRI), Kisumu, Kenya; d Division of Global Health Protection, Center for Global Health, Centers for Disease Control and Prevention, Atlanta, GA, USA; e The Emory Global Health Institute, Emory University, Atlanta, GA, USA; f Division of Global Health Protection, Center for Global Health, Centers for Disease Control and Prevention, Nairobi, Kenya

**Keywords:** Mortality rate, cause of death, urban informal settlements, Kibera, Kenya

## Abstract

**Background:**

Reliable mortality data are important for evaluating the impact of health interventions. However, data on mortality patterns among populations living in urban informal settlements are limited.

**Objectives:**

To examine the mortality patterns and trends in an urban informal settlement in Kibera, Nairobi, Kenya,

**Methods:**

Using data from a population-based surveillance platform we estimated overall and cause-specific mortality rates for all age groups using person-year-observation (pyo) denominators and using Poisson regression tested for trends in mortality rates over time. We compared associated mortality rates across groups using incidence rate ratios (IRR). Assignment of probable cause(s) of death was done using the InterVA-4 model.

**Results:**

We registered 1134 deaths from 2009 to 2018, yielding a crude mortality rate of 4.4 (95% Confidence Interval [CI]4.2–4.7) per 1,000 pyo. Males had higher overall mortality rates than females (incidence rate ratio [IRR], 1.44; 95% CI, 1.28–1.62). The highest mortality rate was observed among children aged < 12 months (41.5 per 1,000 pyo; 95% CI 36.6–46.9). All-cause mortality rates among children < 12 months were higher than that of children aged 1–4 years (IRR, 8.5; 95% CI, 6.95–10.35). The overall mortality rate significantly declined over the period, from 6.7 per 1,000 pyo (95% CI, 5.7–7.8) in 2009 to 2.7 (95% CI, 2.0–3.4) per 1,000 pyo in 2018. The most common cause of death was acute respiratory infections (ARI)/pneumonia (18.1%). Among children < 5 years, the ARI/pneumonia deaths rate declined significantly over the study period (5.06 per 1,000 pyo in 2009 to 0.61 per 1,000 pyo in 2018; *p* = 0.004). Similarly, death due to pulmonary tuberculosis among persons 5 years and above significantly declined (0.98 per 1,000 pyo in 2009 to 0.25 per 1,000 pyo in 2018; *p* = 0.006).

**Conclusions:**

Overall and some cause-specific mortality rates declined over time, representing important public health successes among this population.

## Introduction

Reliable and timely mortality data are important for health services planning, prioritisation and evaluating the impact of health interventions [1]. Monitoring levels and trends in cause-specific mortality can provide critical insights into emerging or neglected health problems [2,3]. However, only 58% of deaths were registered globally in 2015 and only 40% of countries registered at least 90% of deaths in 2020 [4,5]. Systematic health and vital event surveillance systems are weakest in countries with limited resources and with the highest mortality burdens [6,7]. Resource-poor-settings often lack accurate, complete and timely reporting of in-hospital deaths [8,9]. Deaths occurring outside health facilities are even less well counted, with causes of death frequently undocumented [7]. The likelihood of a death being registered and having a documented cause depends strongly on the socioeconomic status of the community and nation in which it occurs [10].

Globally more than half of the world’s population live in urban areas, and the urban population is growing most rapidly in Asia and Africa [11]. In sub-Saharan Africa in 2018, 54% of the urban population lived in informal settlements, a proportion considerably higher than the global average of 29% [12]. Informal urban settlements are generally characterised by high population density, inadequate housing, poor sanitation, limited access to clean water, and poverty – conditions which adversely impact the health of community residents [13]. In addition, access to health care within these settlements is often very limited [14]. Despite the sizeable population residing in urban informal settlements in sub-Saharan Africa and the unfavourable health conditions therein, limited data are available on rates and causes of mortality in this context [15]. Using data from population-based mortality surveillance and verbal autopsy (VA) data collected over a 10-year period in Kibera, Kenya, we sought to bridge the information gap on mortality patterns in urban informal settlements.

## Methods

### Study site and population

The Kenya Medical Research Institute (KEMRI) in collaboration with the US Centers for Disease Control and Prevention (CDC) has implemented the Population-Based Infectious Disease Surveillance (PBIDS) platform since 2006 in Kibera, a large urban informal settlement in Nairobi, Kenya. The surveillance methods have been described elsewhere [16,17]. Kibera is densely populated, with inadequate sanitation and clean water and a high prevalence of human immunodeficiency virus (HIV) infection (12% among adults in 2012) [18]. A majority of the residents are Luo, an ethnic community native to western Kenya [19]. Malaria is not endemic in Kibera, but residents frequently travel to rural areas of the country, especially the lake region in western Kenya, where malaria is endemic [20]. Vaccines against *Hemophilus influenzae* type B, *Streptococcus pneumoniae* and rotavirus were introduced in the routine childhood immunisation programme in Kenya in 2001, 2011 and 2014 respectively. In addition, a cross-sectional study conducted among residents of Kibera who delivered in 2014–2015 reported that 97% had delivered in a health facility. Individuals residing in a defined catchment area within Kibera for at least 4 months are eligible for participation in PBIDS. Infants born to PBIDS participants are also eligible, regardless of the duration of residence. A person stops being a resident if reported to be away for more than 4 months. During the study period, the population under surveillance ranged from approximately 22,000 to 28,000. PBIDS participants receive free health care for acute infectious illnesses at a centrally located clinic where facility-based surveillance for infectious diseases is also conducted.

### Data sources

Surveillance is conducted at the household level, with regular visits by trained study personnel using standardised questionnaires to collect demographic data, including births, deaths, and migrations of household members. Starting in January 2006, home visits were conducted every 2 weeks; in October 2009, the frequency was increased to weekly, and then returned to biweekly in May 2011. In April 2015, the frequency of household visits decreased to every 6 months. The key demographic data gathered within a household did not change over the surveillance period. Since decreasing the frequency of household visits in 2015, household data collection has been supplemented by a network of community reporters; residents of the study area who provide continuous reports of pregnancies, births and deaths as they occur.

Verbal autopsy (VA) is attempted for all reported deaths within a month after death by specially trained field workers who interview the deceased’s next of kin, or health workers who cared for the person at home or are familiar with the circumstances of the death. VA interviews are conducted using standard questionnaires based on tools from the World Health Organization (WHO), with updates to new versions in 2013 (2012 WHO VA questionnaire) and 2018 (2016 WHO VA questionnaire) [21].

### Statistical analyses

Data management and analyses were performed in STATA Version 13.1 software (Stata Corp., College Station, TX, USA) and R for Windows version 3.6.1. We analysed all deaths occurring among PBIDS participants of all ages between January 2009 and December 2018. Assigning of probable cause(s) of death was done using a Bayesian probabilistic model, the InterVA-4 model version 4.02, setting HIV prevalence to high and malaria prevalence to low. The causes of death generated by InterVA-4 are compatible with the International Classification of Diseases version 10 (ICD-10) and are categorised into 62 groups, as defined in the WHO VA instrument [22]. The specific causes were grouped, based on inter-VA model, into communicable disease, non-communicable disease, maternal, neonatal and injuries [23]. Maternal and neonatal categories included communicable or non-communicable causes within these categories.

Results are presented as annualised mortality rates and fractions. All-cause, cause-specific, communicable, and non-communicable disease mortality rates were expressed as the number of deaths per 1,000 person-years of observation (pyo), calculated based on each PBIDS participant’s residency status during the study period. Cause-specific and grouped cause-specific mortality rates were adjusted for the proportion of deaths missing VA by dividing the number of deaths due to a particular cause by the proportion of all deaths with VA conducted to obtain an adjusted number of cause-specific deaths; adjustments were made stratified by sex, age groups and year. Deaths classified as indeterminate were excluded from cause- and group-specific rates.

Assuming a Poisson distribution, mortality rates with 95% CI were estimated and compared across groups using incidence rate ratios (IRR). We used Poisson regression to model for trends in mortality rates over time using log_e_ (person – time) as offset and categorical year as the exposure variable. A statistically significant change was set at *p* < 0.05.

## Results

### All-cause mortality

A total of 1134 deaths were registered from January 2009 to December 2018 of which 774 (68.2%) had complete VA conducted. Causes of death were available for 735 (95.0%) of the completed deaths with VA. Of those without cause of death 16 (41.0%) were female and 23 (58.9%) were male. There were 256,445 pyo during the study period (Table 1). The overall all-cause mortality rate was 4.4 per 1,000 pyo (95% confidence interval [CI]; 4.2–4.7). The highest mortality rate was observed among children aged <12 months (41.5 per 1,000 pyo; 95% CI 36.6–46.9) and persons aged ≥65 years (32.6 per 1,000 pyo; 95% CI, 21.5–47.5) and lowest in persons aged 5–14 years (1.1 per 1,000 pyo; 95% CI, 0.9–1.4). Overall mortality rate among children <12 months was more than eight times that of children aged 1–4 years (incidence rate ratio (IRR), 8.5; 95% CI, 6.95–10.35; *p* < 0.001). The overall mortality rate in males (5.2 [95% CI, 4.8–5.7]) was higher than in females 3.6 (95% CI, 3.3–4.0) per 1,000 pyo.

**Table 1.** Number of deaths, proportion of deaths with verbal autopsy and all-cause mortality rates by age, sex, and year in Kibera, 2009–2018.

Characteristics	No. of deaths	PYO*	Mortality rates (95% CI)	No. of VA^¥^ done		Determinate CoD^±^	
Age group				n	%	n	%
<1	260	6263.1	41.5 (36.6–46.9)	198	76.2	174	87.9
1–4	155	31695.9	4.9 (4.2–5.7)	104	67.1	101	97.1
5–14	82	73523.5	1.1 (0.9–1.4)	51	62.2	48	94.1
15–49	507	134988.5	3.8 (3.4–4.1)	331	65.3	323	97.6
50–64	103	9147.0	11.3 (9.2–13.7)	68	66.0	67	98.5
65+	27	827.4	32.6 (21.5–47.5)	22	81.5	22	100.0
Sex							
Female	478	131306.0	3.6 (3.3–4.0)	318	66.5	302	95.0
Male	656	125139.5	5.2 (4.8–5.7)	456	69.5	433	95.0
Year							
2009	173	25792.3	6.7 (5.7–7.8)	98	56.6	96	98.0
2010	185	28099.7	6.6 (5.7–7.6)	106	57.3	101	95.3
2011	156	27811.8	5.6 (4.8–6.6)	110	70.5	99	90.0
2012	125	27033.2	4.6 (3.8–5.5)	91	72.8	84	92.3
2013	111	26570.2	4.2 (3.4–5.0)	74	66.7	71	95.9
2014	111	26910.6	4.1 (3.4–5.0)	92	82.9	86	93.5
2015	70	24173.5	2.9(2.3–3.7)	49	70.0	48	98.0
2016	57	23638.6	2.4 (1.8–3.1)	39	68.4	37	94.9
2017	84	23193.1	3.6 (2.9–4.5)	70	83.3	68	97.1
2018	62	23222.4	2.7 (2.0–3.4)	45	72.6	45	100.0
All	1134	256445.4	4.4 (4.2–4.7)	774	68.3	735	95.0

*PYO; Person years of observation; CI: Confidence interval; ^¥^VA: Verbal autopsy: ^±^CoD: Cause of Death.

**Figure 1.**
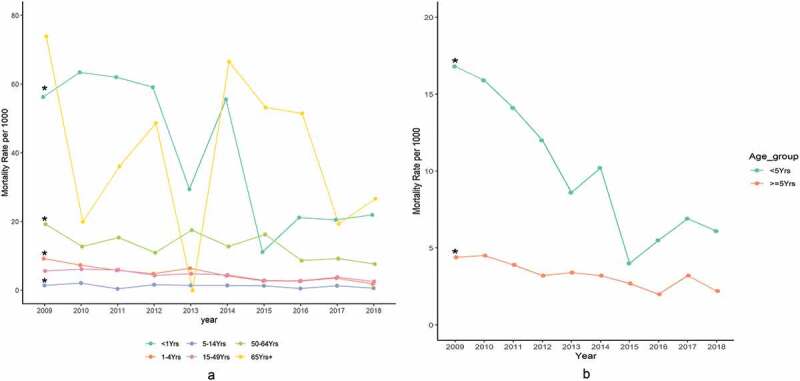
(a) All-cause mortality rates per 1,000 pyo by age group in Kibera, Kenya, 2009 to 2018. *Significant reduction at *p* < 0.05. (b) All-cause mortality rates per 1,000 pyo by age group in Kibera PBIDS, Kenya, 2009–2018. *Significant reduction at *p* < 0.05.

The overall mortality rate decreased from 6.7 per 1,000 pyo (95% CI, 5.7–7.8) in 2009 to 2.7 (95% CI, 2.0–3.4) per 1,000 pyo in 2018. Significant declines were observed among children aged <5 years, with mortality falling from 16.8 (95% CI 13.3–20.8) per 1,000 pyo in 2009 to 6.1 (95% CI, 3.5–9.7) per 1,000 pyo in 2018 (*p* < 0.001) (Figure 1(a,b)). Although the mortality rate among children aged <1 year was consistently higher than that among those aged 1–4 years), the % reduction in both groups was similar (13% and 15% respectively). A significant decline over time was also seen in persons aged ≥5 years; mortality fell from 4.4 (95% CI 3.5–5.4) per 1,000 pyo in 2009 to 2.2 (95% CI 1.6–2.9) per 1,000 pyo in 2018; *p* < 0.001. Significant decreases in mortality were observed in both females (5.5 per 1,000 pyo in 2009 to 1.8 per 1,000 pyo in 2018; *p* < 0.001) and males (7.9 per 1,000 pyo in 2009 to 3.5 per 1,000 pyo in 2018; *p* < 0.001). Overall, male had higher mortality rates than females (IRR, 1.44; 95% CI, 1.28–1.62; *p* < 0.001).

### Cause specific mortality

**Figure 2.**
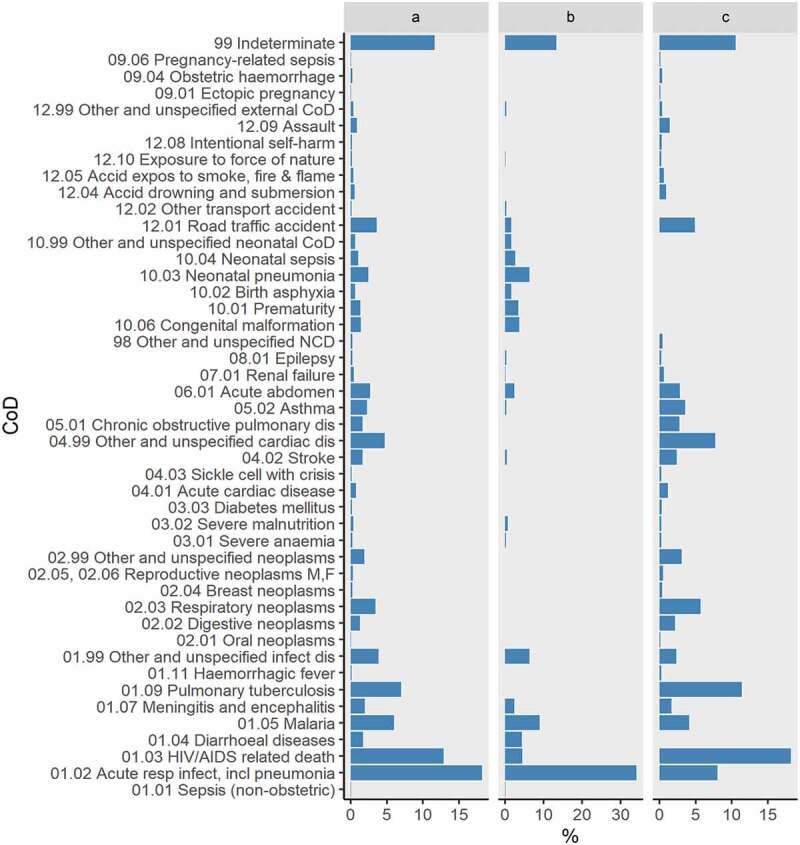
Generalized causes of death among all age groups (a) children aged <5 years (b) and persons aged ≥5 years (c) in Kibera, Kenya,2009–2018.CoD = Cause of death; NCD = non-communicable diseases.

**Figure 3.**
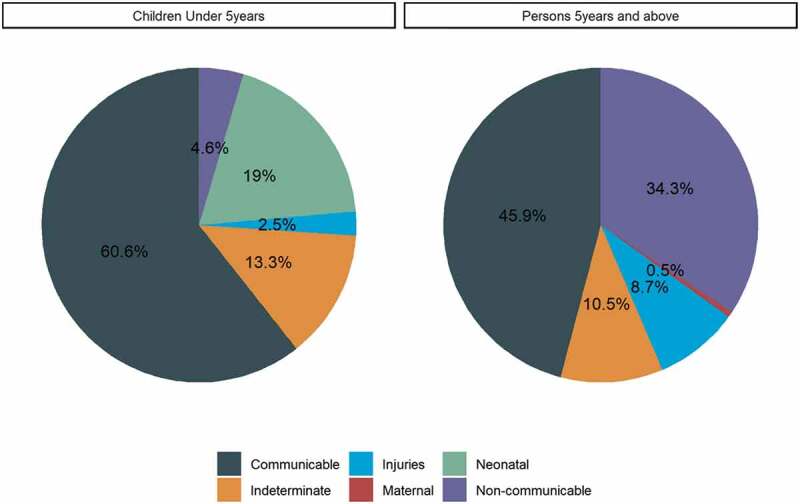
Overall grouped cause of death by age group in Kibera PBIDS, Kenya,2009–2018.

Among 774 deaths with VA, the most common cause of death was acute respiratory infections/pneumonia (18.1%), followed by HIV/AIDS related deaths (12.8%), pulmonary tuberculosis (6.9%) and malaria (5.9%); of note, 11.6% had an indeterminate cause (Figure 2). In children aged <5 years, acute respiratory infections/pneumonia (34.0%) was most frequent, followed by malaria (8.7%) and neonatal pneumonia (6.3%). In persons aged ≥5 years, the most common causes were HIV/AIDS related deaths (18.2%), pulmonary tuberculosis (11.4%), acute respiratory infection/pneumonia (8.0%) and unspecified cardiac disease (7.7%) (Figure 2). On grouping the causes of deaths (Figure 3), communicable diseases (60.6%) were most common among children aged <5 years followed by neonatal causes (19.0%). Among persons aged ≥5 years, communicable diseases (45.8%) were also most frequent, followed by non-communicable diseases (34.4%) and injuries (8.7%). Overall, the leading COD among non-communicable diseases was respiratory neoplasms (3.5%).

**Figure 4.**
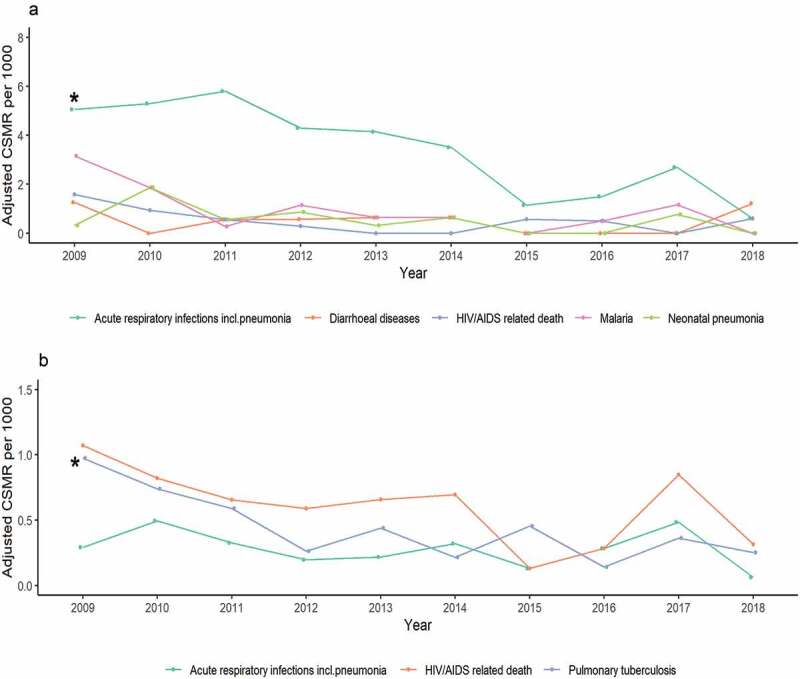
Adjusted cause-specific mortality rate for leading CoD by age group in Kibera PBIDS, Kenya,2009–2018. (a) Children under 5 years; (b) Persons 5 years and above; CSMR: Cause specific mortality rate. *Significant reduction at *p* < 0.05.

Cause-specific mortality rates over time for the leading causes of death are presented in Figure 4. Among children aged <5 years, the rate of acute respiratory infection/pneumonia deaths declined significantly over the study period (5.06 per 1,000 pyo in 2009 to 0.61 per 1,000 pyo in 2018; *p* = 0.004). The rates of malaria, diarrhoea, HIV/AIDS and neonatal pneumonia deaths in children aged <5 years did not significantly change over time. Among persons aged ≥5 years, HIV/AIDS related deaths remained the highest cause-specific rate throughout most years of the study period. HIV/AIDS mortality rate was highest in 2009 (1.07 per 1,000 pyo) and lowest in 2015 (0.13 per 1,000 pyo); however, there was not a statistically significant decline over the study period. The rate of death due to pulmonary tuberculosis among persons aged ≥5 years declined significantly (0.98 per 1,000 pyo in 2009 to 0.25 per 1,000 pyo in 2018; *p* = 0.006).

**Figure 5.**
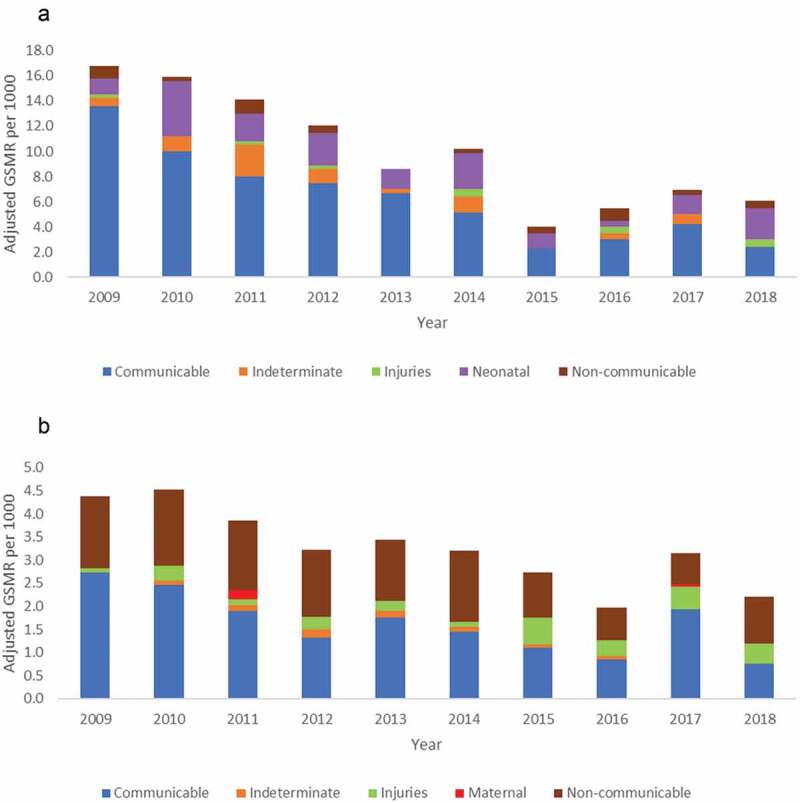
Adjusted group-specific mortality rate by year in Kibera PBIDS, Kenya,2009–2018. (a) Children under 5 years; (b) Persons 5 years and above; GSMR: Group-specific mortality rate.

Trends in group-specific mortality (communicable, non-communicable, maternal, neonatal, and injury) are shown in Figure 5. Mortality due to communicable diseases declined significantly among both those aged <5 years (13.6 per 1,000 pyo in 2009 to 2.4 per 1,000 pyo in 2018; *p* < .001) and those aged ≥5 years (2.7 per 1,000 pyo in 2009 to 0.8 per 1,000 pyo in 2018; *p* < .001). Mortality due to other grouped causes of death (non-communicable, neonatal, maternal and injury) did not significantly change over time.

## Discussion

Over a 10-year period, the overall mortality rate declined significantly in Kibera, with a decrease of ~64% in children aged <5 and ~50% among older children and adults. The observed reduction was driven primarily by declines in deaths due to infectious causes, including acute respiratory infection (ARI)/pneumonia in children aged <5 years and tuberculosis (TB) in persons aged ≥5 years. Despite this progress, we observed a persistent burden of deaths due to infectious diseases such as HIV/AIDS and pneumonia in adults, and malaria, diarrhoea and HIV/AIDS in young children. We also observed a substantial variability in risk of death across demographic groups, highlighting the importance of mortality data for guiding public health interventions.

We found ARI/pneumonia to be the predominant cause of death among children aged <5 years and a leading cause of mortality among older children and adults. Several studies have similarly highlighted respiratory infections as a leading cause of morbidity and mortality in informal settlements [24,25]. Factors such as indoor air pollution and overcrowding, which are a characteristic of informal settlements, favour spread of respiratory pathogens [26,27]. Over the 10-year study period, we observed an 88% decline in the rate of pneumonia/ARI deaths among children aged <5 years. The introduction of the 10-valent pneumococcal conjugate vaccine (PCV10) in Kenya in January 2011 likely contributed to that reduction particularly for vaccine-specific serotypes. A study of PCV10 impact in Brazil observed a decline of ~10% in childhood pneumonia mortality attributable to the vaccine, with greater reductions in lower socioeconomic regions [28]. A study in South Africa reported a decline in pneumonia mortality attributable to PCV (7- and 13-valent formulations) of 33% and 23% in children aged 1–11 months and 1–4 years respectively [29]. That study also reported an indirect effect of PCV on pneumonia morality among children aged 8–18, with a reduction of 23% among this group too old for PCV vaccination. We did not observe a significant decline in pneumonia/ARI mortality among persons aged >5 years, nor was there a decline in neonatal pneumonia. Furthermore, despite the substantial reduction of ARI/pneumonia deaths among children aged <5 years, it remained a leading cause of mortality among this group even in the latter years of the study. Additional interventions such as improving housing conditions and reducing indoor air pollution, as well as vaccines against important respiratory pathogens such as the respiratory syncytial virus, currently under development, are needed to further reduce the pneumonia mortality burden in Kibera. HIV/AIDS and TB were leading causes of death among persons aged ≥5 years, with a significant decline in TB deaths and no significant change in HIV/AIDS deaths over the study period. Other studies have similarly reported HIV/AIDS and TB as major causes of adult deaths in sub-Saharan Africa, particularly among people living in informal settlements [30,31]. In Kenya, HIV/AIDS prevention and control efforts have led to reductions in disease burden; national adult HIV prevalence rate fell from 6.0% in 2006 to 4.9% in 2017, when adult ART coverage was estimated at 75% [32]. Despite these efforts, the burden of HIV/AIDS remains relatively high in urban informal settlements, which may be related to risky sexual practices such as early sexual debut among residents in urban informal settlements [18,33]. The high TB burden in urban informal settlements is likely related to both HIV prevalence in this setting, as well as high population density, which promotes the spread of respiratory pathogens. Further reduction in HIV/AIDS and TB mortality in urban informal settlements will likely require multi-faceted interventions that address the root causes of the high burdens in these settings.

Malaria stood out as an important cause of death among children aged <5 years despite Nairobi being considered a low-risk area for malaria transmission [34]. A study conducted among PBIDS participants in Kibera from 2007 to 2011 found that among febrile patients presenting to an outpatient clinic with microscopy performed, 22% had malaria parasites detected, including 23% among those aged <5 years; 64% of malaria cases reported travel outside Nairobi in the past month; 79% of these had travelled to three counties in western Kenya with a very high malaria burden [35]. Therefore, malaria deaths occurring in Kibera most likely reflect infections acquired outside of Nairobi. The InterVA model has only two settings for malaria (high or low); we set malaria prevalence to low (<1%) based on lack of evidence of transmission of malaria in Nairobi. However, it is possible that some deaths due to other febrile illnesses were misclassified by the InterVA model as attributable to malaria, resulting in an overestimation of malaria deaths. On the other hand, the model does not account for travel to high malaria endemic areas, which could lead to an underestimation of malaria deaths. Refinements to InterVA may be warranted to improve the accuracy of results in areas with varying levels of malaria endemicity. Nonetheless, this study has highlighted the need for clinicians to consider malaria as a potential cause of illness among children, even in low-risk and non-transmission areas.

Injuries and other non-communicable diseases were major contributors to mortality among persons aged >5 years, with no significant change over time. This is contrary to other studies which have reported increases in deaths due to injuries and non-communicable diseases among adults living in informal settlements [36–38]. Even without a significant increase in deaths due to injuries and non-communicable diseases, these causes become increasingly important as deaths due to communicable diseases decline. Risk factors for non-communicable diseases – including unhealthy diet, sedentary lifestyle, harmful alcohol consumption and tobacco use – have been on the rise in urban informal settlements in Africa [39]. Respiratory neoplasm was a major contributor to mortality related to NCDs among older children and adults. These deaths combined with high number of ARI/pneumonia related deaths may suggest household air pollution and smoking as important risk factors in this setting. Several studies conducted to assess air quality have reported high levels of fine particulate matters (PM_2.5_) in similar settings in Kenya [40,41]. Efforts to reduce mortality in urban informal settlements will require effective evidence-informed policies to address prevention and control of injuries and other non-communicable disease.

This study had several limitations. It is important to note that misclassification errors do occur in all forms of cause-of-death assignment, including the use of InterVA-4 modelling and that InterVA-4 provides only a single cause of death per individual. Furthermore, 32% of reported deaths in our study had no VA interviews conducted primary due to lack of appropriate respondent, refusal to participate in the VA interviews and migration of family members of the deceased. Since we lack information on the causes of death in this particular group with missing VA there is a risk of underestimation of the disease burden in this population. To try and mitigate this bias we adjusted for missing VA in calculating cause-specific mortality rates. Of note, PBIDS participants receive health care for acute illnesses free of charge at the surveillance clinic, and this access to care might have impacted mortality rates over time.

## Conclusion

Despite these limitations, we emphasise the important role of verbal autopsy data in filling the gap created by inadequate vital registration systems in this setting. VA can be used to monitor population-level cause of death over time. We found that overall all-cause mortality rates and deaths due to specific infectious causes declined over time in Kibera, representing important public health successes among this underserved urban population. However, communicable diseases persist as leading causes of death, despite the availability of preventive interventions. In addition, rates of death due to injuries and non-communicable diseases have remained stable over time, and therefore responsible for an increasing proportion of death, particularly among persons aged ≥5 years. Current preventive strategies for infectious diseases and non-communicable diseases may need to be tailored to the informal settlement setting to maximise impact on mortality.

## Supplementary Material

Supplemental Material

## Paper context

Despite the sizeable population residing in urban informal settlements in sub-Saharan Africa (SSA) and the unfavourable health conditions therein, limited data are available on rates and causes of mortality in this context. The analysis presented here highlights the mortality patterns and trends within this underserved urban population. We hope that public health experts and policy makers interested in improving health outcomes within urban informal settlements in SSA will find this paper of interest.
