# Supplementary material for: Mortality patterns over a 10-year period in Kibera, an urban informal settlement in Nairobi, Kenya, 2009–2018
Source: Glob Health Action. 2023 Jul 25;16(1):2238428. doi: 10.1080/16549716.2023.2238428 (PMC10392302; doi:10.1080/16549716.2023.2238428)
Supplement: Supplemental Material [file ZGHA_A_2238428_SM9885.docx]

Supplementary table 1: Number of deaths, proportion of deaths with and without verbal autopsy conducted by age, sex, and year in Kibera, 2009 – 2018.

| Characteristics | Total deaths | With VA | % | Without VA | % |
| --- | --- | --- | --- | --- | --- |
| Age Group |  |  |  |  |  |
| <1 | 260 | 198 | 25.6 | 62 | 17.2 |
| 1–4 | 155 | 104 | 13.4 | 51 | 14.2 |
| 5–14 | 82 | 51 | 6.6 | 31 | 8.6 |
| 15–49 | 507 | 331 | 42.8 | 176 | 48.9 |
| 50–64 | 103 | 68 | 8.8 | 35 | 9.7 |
| 65+ | 27 | 22 | 2.8 | 5 | 1.4 |
| Sex |  |  |  |  |  |
| Female | 478 | 318 | 41.1 | 160 | 44.4 |
| Male | 656 | 456 | 58.9 | 200 | 55.6 |
| Year |  |  |  |  |  |
| 2009 | 173 | 98 | 12.7 | 75 | 20.8 |
| 2010 | 185 | 106 | 13.7 | 79 | 21.9 |
| 2011 | 156 | 110 | 14.2 | 46 | 12.8 |
| 2012 | 125 | 91 | 11.8 | 34 | 9.4 |
| 2013 | 111 | 74 | 9.6 | 37 | 10.3 |
| 2014 | 111 | 92 | 11.9 | 19 | 5.3 |
| 2015 | 70 | 49 | 6.3 | 21 | 5.8 |
| 2016 | 57 | 39 | 5.0 | 18 | 5.0 |
| 2017 | 84 | 70 | 9.0 | 14 | 3.9 |
| 2018 | 62 | 45 | 5.8 | 17 | 4.7 |
